# Luminescence Properties of Nano Zinc Oxide Doped with Al(III) Ions Obtained in Microwave-Assisted Hydrothermal Synthesis

**DOI:** 10.3390/ma15041403

**Published:** 2022-02-14

**Authors:** Tomasz Strachowski, Ewa Grzanka, Jan Mizeracki, Adrian Chlanda, Magdalena Baran, Marcin Małek, Klaudia Onyszko, Bartosz Januszewski, Mirosław Przybysz

**Affiliations:** 1 Research Group of Graphene and Composites, Łukasiewicz Research Network–Institute of Microelectronics and Photonics IMiF, Al. Lotnikow 32/46, 02-668 Warsaw, Poland; adrian.chlanda@imif.lukasiewicz.gov.pl (A.C.); magdalena.baran@imif.lukasiewicz.gov.pl (M.B.); 2 Institute of High Pressure Physics PAS “Unipress”, Sokolowska 29/37, 01-142 Warsaw, Poland; elesk@unipress.waw.pl (E.G.); janekm@unipress.waw.pl (J.M.); 3 Faculty of Civil Engineering and Geodesy, Military University of Technology, ul. Gen. Sylwestra Kaliskiego 2, 00-908 Warsaw, Poland; marcin.malek@wat.edu.pl (M.M.); klaudia.onyszko@wat.edu.pl (K.O.); bartosz.januszewski@wat.edu.pl (B.J.); 4 Institute of Robots Machine Design, Faculty of Mechanical Engineering, Military University of Technology, ul. Gen. Sylwestra Kaliskiego 2, 00-908 Warsaw, Poland; miroslaw.przybysz@wat.edu.pl

**Keywords:** doped zinc oxide, luminescence properties, microwave-assisted synthesis, hydrothermal synthesis

## Abstract

The hydrothermal method of obtaining nano zinc oxide doped with different contents of aluminum ions (III) was presented and discussed in this paper. Aqueous solution of Zn(NO_3_)_2_*6H_2_O and Al(NO_3_)_3_*9H_2_O salts mixture were used as the synthesis precursor. In order to reduce the process time all reactions were performed in a microwave reactor. The influence of process parameters and the content of impurity ions on the properties of synthesized nano zinc oxide were analyzed. In addition to zinc oxide doped with Al(III) ions, an additional spinel phase (ZnAl_2_O_4_) was obtained. The luminescent properties of nano zinc oxide as a function of the dopant ions were also discussed. Based on the luminescence measurements results, it was found that the luminescence intensity decreases with the increasing dopant content. The obtained materials are aimed to be implemented as luminescent materials in optoelectronic and sensors.

## 1. Introduction

The production of nanometer-sized powders and their application in innovative technologies is an important and growing area of interest of both scientific and economy societies. Previous research in this area has shown that shape and size distribution of the particles can strongly influence the properties of resulting nanopowders. The chemistry of the compounds also plays an important role, determining some specific chemical-related properties of the surface. Depending on these properties it is possible to attach specific chemical groups (surface functionalization) or to introduce ions of various chemical elements into the crystallographic lattice.

Nano zinc oxide is a chemical compound with unique physical and chemical properties. It belongs to the amphoteric oxides. Due to its properties, it has become a material willingly used in many branches of industries, such as: rubber [1], pharmaceutical [2,3,4], dye [5,6,7], ceramic [8,9], and the chemical industry [10].

It is also used for production of varistors [11,12], sensors [13,14,15], semiconductors and due to a wide energy gap (3.37 eV) in optoelectronics [16,17,18,19,20,21,22]. Doping of such material with aluminum ions leads to another applications. Doped material is used for the production of thin layers, which are subsequently used in the manufacturing process of solar cells. When embedded in a polyester matrix, they can be also used for production of polymeric displays in various types of devices [23]. Thin layers of nano zinc oxide doped with different ions can be obtained by the sol-gel method [24,25,26,27,28] and by vacuum condensation [29,30,31,32]. In addition, zinc oxide doped with Al(III) ions can also be obtained by a hydrothermal method using various precursors [33,34,35,36,37,38]. 

The main motivation for this research was the authors’ long experience in the synthesis of zinc oxide doped with Al(III) ions. The syntheses were carried out by the classical hydrothermal method in an autoclave [36,38] and by gas condensation [29]. Even if successful, such an approach was time consuming, thus we decided to introduce a microwave reactor to produce zinc oxide doped with Al(III) ions of at least the same quality, as when using aforementioned methods. In addition to zinc oxide doped with Al ions, an additional spinel phase ZnAl_2_O_4_ was obtained. It was formed when the dopant ion content was around 15%. One can find literature reports describing the utilization of zinc-oxide particles towards different applications [39,40,41]. Compared with the results presented in the aforementioned papers, materials synthesized in this study were characterized with a smaller grain size, which could trigger a different biological response from the resulting material. What is more, the synthesis protocol implemented in this study was described by a shorter process time, a lower temperature, and lower microwave power in comparison with the cited works. The present work is focused on the preparation of nano zinc oxide doped with aluminum ions by a hydrothermal method using the microwave reactor.

## 2. Materials and Methods

### 2.1. Hydrothermal Synthesis of Nano Zinc Oxide Doped with Al(III) Ions

The Ertec microwave reactor (Ertec Poland, Wrocław, Poland) [42] is a laboratory instrument designed for hydrothermal synthesis in a microwave field. This reactor consists of a pressure head and a Teflon reaction vessel with a sealed lid and a pressure diaphragm as a burstable pressure fuse. Microwave energy is drawn into the head from the magnetron through a waveguide in which a short antenna is immersed. The whole system is controlled by means of a CPU (Central Processing Unit) controller, communicating with the PC (Personal Computer). The computer program allows adjusting power thresholds and pressure limits, along with temperature and pressure registration during the process [43,44,45]. 

The hydrothermal synthesis was carried out in the microwave reactor (Ertec). Appropriate amounts of zinc nitrate (Sigma-Aldrich, 99%) and aluminum nitrate (Sigma-Aldrich, 99%) were added to a distilled water in order to obtain a solution of 1 M concentration. The content of aluminum ions ranged from 0.2 to 15% molar. The next step was to add an aqueous solution of potassium hydroxide (2M) (Sigma-Aldrich, 98%), to raise the pH of the zinc-aluminum salt solution to 9–10 and precipitate the corresponding zinc and aluminum hydroxides. The solutions (without leaching the remaining ions) were then poured into a Teflon reaction vessel (Teflon, Wilmington, DE, US) (110 ml capacity), which was then placed in a microwave reactor and the syntheses were performed. Process parameters included inter alia: process time (heating and cooling time), maximum temperature and pressure. These parameters are of crucial importance and must be precisely determined in order to obtain a good quality material. The process started at room temperature, followed by heating to 120℃ at a rate of 20 °C/min. Subsequently, the hydrogel was kept under specified conditions for: 5, 10 and 15 min. Next, the reactor was cooled down and opened. The resulting product was filtered using filter media and was washed repeatedly with distilled water. It was then dried in a vacuum dryer for 24 h at a temperature of 70 °C.

### 2.2. Methods

Siemens D-5000 X-ray diffractometer (Siemens/Bruker, Munich, Germany) was used to analyze the obtained synthesis products. It enabled the determination of phase composition, recognition of crystallographic lattice and coordinates of atoms in the elementary cell (by Rietvield method) [46]. 

The helium density was determined by gas pycnometry with the Accupyc 1330 helium pycnometer (Micromeritics,Norcross, GA, US). The specific surface area was measured using BET adsorption method with a Micromeritics device (Micromeritics, Norcross, GA,US) [47]. 

Microstructure studies of nanopowders were performed using scanning microscopy method with a Zeiss LEO1530 (Zeiss, Oberkochen, Germany) equipped with Zeiss Gemini column. Photoluminescence measurements were performed at a room temperature using a Spectrofluorimeter CM2203 (SOLAR, Minsk, Belarus). Both slits of the monochromator, the excitation channel and the detection channel, were set at an optical width of 5 nm. All samples were excited with a wavelength of 300 nm. The range of photoluminescence measurement was from 340 nm to 820 nm.

## 3. Results and Discussion 

The ceramic preforms were tested using XRD analysis. The material obtained in the microwave reactor was analyzed in order to verify whether the desired product was synthesized. In order to ensure repeatability of experimental data, each experiment was repeated five times. The same amount of material (1 g) was used for density and specific surface area measurements. By doing this, we wanted to ensure repeatability of the experimental procedure. Table 1 presents the results of the specific surface area analysis, density and grain size. At the same time, it is worth noting that the only puzzling result was the low density of the sample with 5% Al(III) ions, which was in fact different from the others tested samples. Further studies are needed in order to explain this phenomenon. This is probably related to the presence of a spinel phase (ZnAl_2_O_4_). The grain size was determined based on XRD spectra analysis using well-known, full-width at half maximum algorithm [48]. 

Based on the literature review, we can conclude that our results are in agreement with the literature data. The obtained samples have a similar density, a specific surface area and lattice constants. In many cases, the materials we obtained were characterized by a smaller grain size. The processes described in the literature were carried out using higher temperatures [33,34,35,36,37,38]. By using a microwave reactor, we were able to shorten the process time and run the process at lower temperatures. In brief: microwave power was set to 600 W, temperature was set to 100–120 °C. We want to underline that several syntheses were carried out to confirm the reproducibility of the process. Each synthesis was conducted with the same parameters (time, temperature, and chemical composition). Based on the obtained results (Table 1 and Table 2), we can state, due to the small deviation of the measured values, that the processes should be considered as fully controlled and repeatable.

On the basis of XRD analysis, it was found that the obtained product was ZnO (reference sample) and ZnO doped with aluminum ions. The fact is that the aluminum ions built into the crystallographic lattice was evidenced by the alteration of network parameters. We observed that with the increasing content of the dopant, the lattice parameters decreased. The reasons for such changes may be related to the fact that two separate phases appeared, ZnO and ZnAl_2_O_4_. Table 2 shows values of lattice parameters determined on the basis of XRD analysis. 

**Figure 1:**
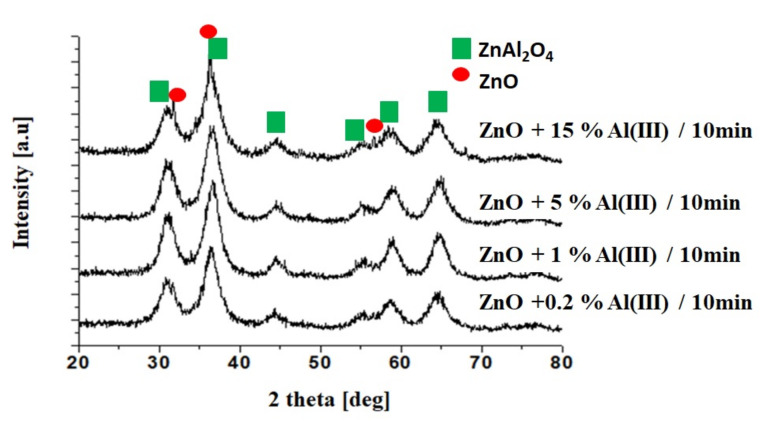
XRD patterns for selected samples obtained in the microwave reactor (10 min).

Figure 1 shows the XRD peaks for selected ZnO samples doped with Al(III) ions obtained in a microwave reactor for 10 min. The appearance of the spinel phase can be observed for samples with an Al(III) ion content of 5 and 15%. Below this value, only the ZnO phase was obtained (Figure S1).

**Figure 2:**
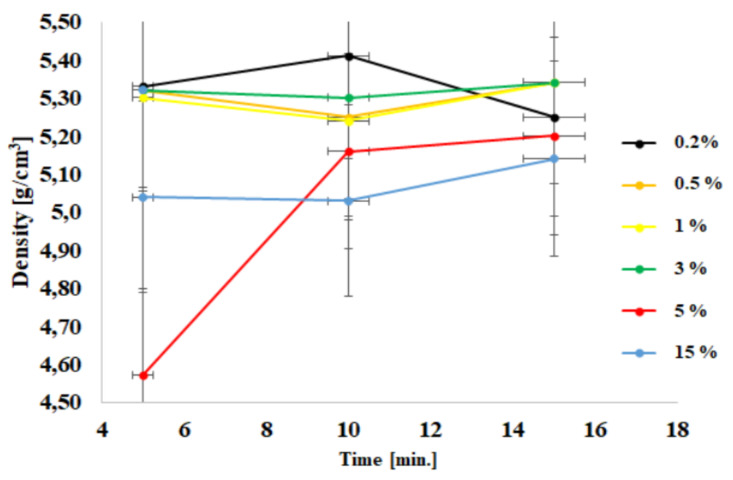
Density dependence as a function of process time.

The diagrams show the dependence of density (Figure 2) and of the specific surface area measured with BET (Figure 2) as a function of the process time. Figure 1 shows the dependence of density on the time of the process and the content of doped ions. It can be observed that apart from the material with a dopant content of 5%, all other materials are characterized with a rather stable density. A drop of this parameter was registered for the sample with the smallest amount of dopant (0.2%).

**Figure 3:**
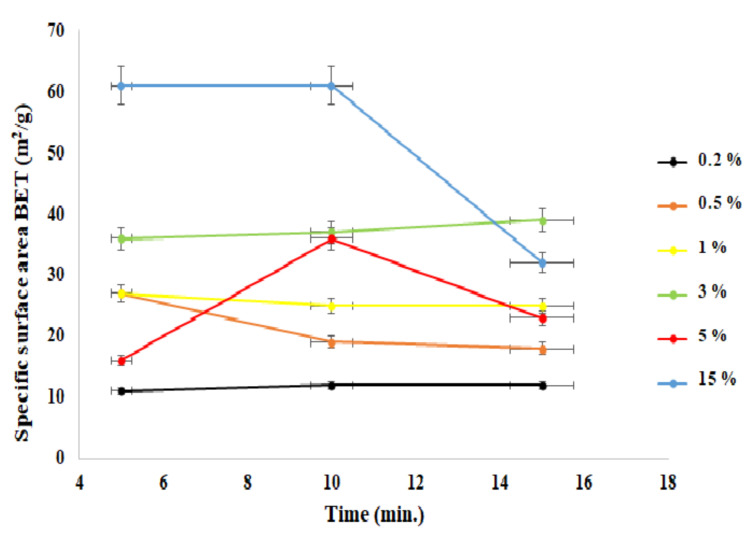
Dependence of the specific surface area (BET) as a function of the process time.

Figure 3 shows the specific surface area as a function of time of the process in the context of doping ions. It was observed that with the increasing level of the doping ions, the specific surface area also increased. It was mostly evidenced for the sample with a content of 15% aluminum ions in the admixture.

**Figure 4:**
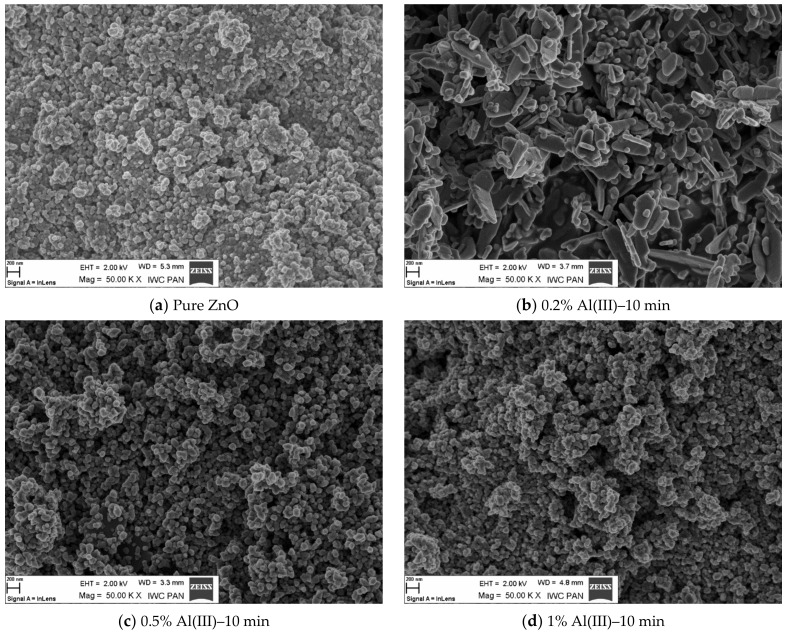

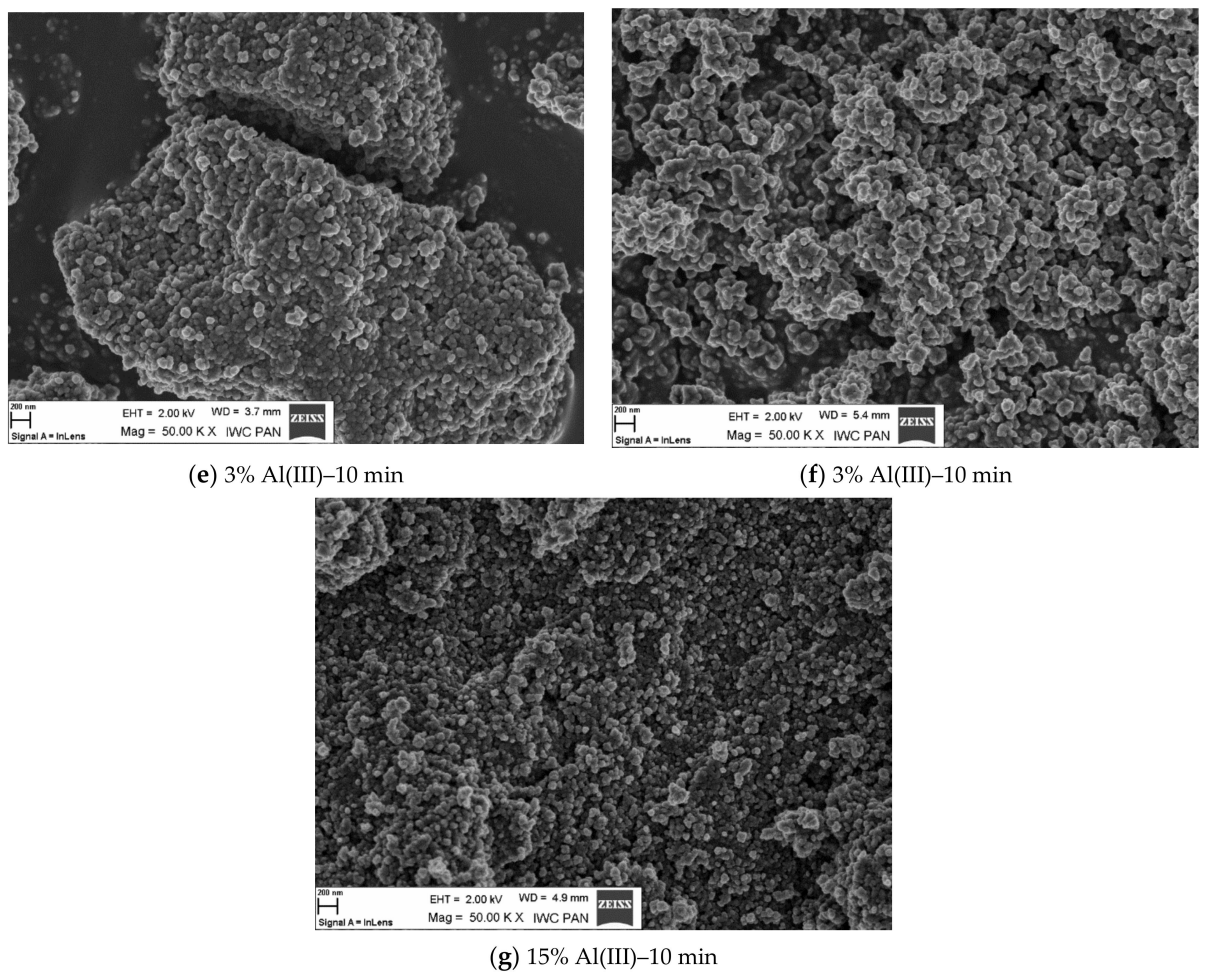
SEM images of zinc oxide morphology doped with Al(III) ions and reference samples.

Figure 4 shows SEM morphological images of nano zinc oxide doped with aluminum ions. The image of pure zinc oxide without any dopant is also presented as a reference.

From the scanning microscope images, dopant ion content-related dependence was deduced. The increasing amount of dopant was reflected in the alteration of morphology of the resulting material. The dopant content altered the specific surface area of the material, as well as the size and shape of the grains [49,50,51,52]. As a result, the obtained material was more agglomerated. This phenomenon could also be influenced by the duration of the fabrication process. The change in morphology as a function of dopant content and process time could have a significant effect on the physical and chemical properties of the resulting material. In the case of presented results, it could affect the luminescence properties. As the dopant content increased, the crystallographic structure was changed. This should be attributed to the appearance of the defects in the structure, which was reflected in the luminescence intensity and the photon emission. The presence of defects is associated with photon emission in the infrared region, which is described in Section Measurement of Luminescence Properties. 

### Measurement of Luminescence Properties

Luminescence—the so-called cold glow—is a physical phenomenon consisting of the emission of electromagnetic radiation characterized by an intensity greater than thermal radiation at a given temperature. It is therefore caused by factors other than the increase in temperature of the emitting source itself. Radiation produced in the luminescence process is barely radiation—a relatively small number of atoms is involved in this phenomenon. Luminescence occurs when the atoms of the medium are excited to states with an energy that is higher than the energy of the ground state. Such atoms then return to their ground or lower excited states by the spontaneous emission of electromagnetic radiation. 

The aim of this study was to characterize photoluminescence (PL) and excitation spectra, as well as to determine the luminescence intensity trend depending on the amount of dopant. For luminescence measurements, a wavelength of 300 nm was chosen as the excitation source in the spectrofluorimeter.

**Figure 5:**
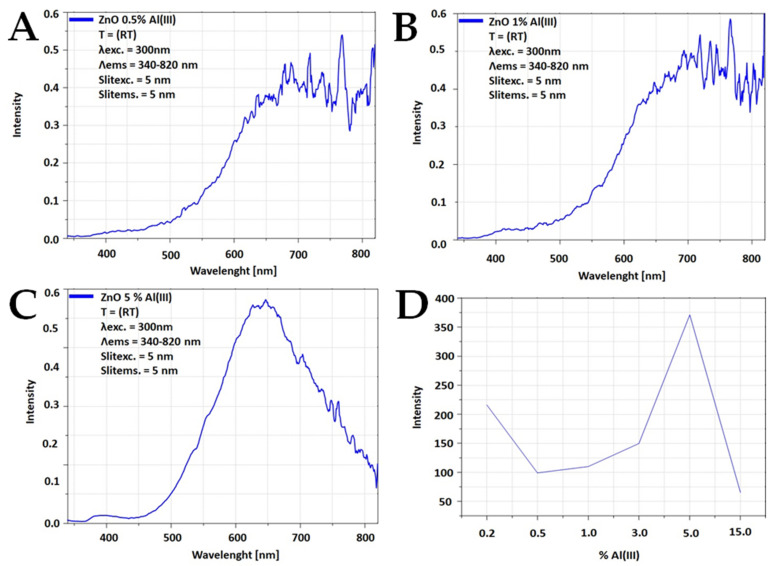
Dependence of luminescence intensity maximum on Al(III) ions content: (**A**) 0.5%, (**B**) 1%, (**C**) 5%, (**D**) maximum intensity.

Figure 5 shows photoluminescence spectra plotted with the amount of doped aluminum ions. Two peaks were observed with excitation at 300 nm. The first maximum was observed at 380 nm and the second maximum was observed at 620 nm. This is a typical luminescence spectrum of ZnO regarding emission in the infrared and UV region [53,54]. Photoluminescence maxima were not observed. This phenomenon could be explained by the fact that low wavelengths resulted in edge illumination, while at higher wavelengths defective illumination was observed. Peaks at longer wavelengths should be treated as an artifact; thus, they were not further analyzed. The measuring system normalized photoluminescence to a constant excitation intensity and amplified the signal for longer wavelengths. Regarding the different positions of the broad photoluminescence, it is a composition of 2–3 broad photoluminescence spectra, which are difficult to separate and thus to analyze.

Compared to the literature, the Al(III) ion-doped zinc oxide we obtained was characterized by a signal at wavelengths above 600 nm. In the literature, one can find reports of a signal in the 400–500 nm region [29,36,49]. 

As the dopant ion content increases, the luminescence intensity decreases. Above 3%, an increase in intensity is observed, which is due to the presence of the spinel phase (ZnAl_2_O_4_).

## 4. Conclusions

This paper presents the results of the synthesis of nano zinc oxide doped with aluminum ions obtained by a hydrothermal method in a microwave reactor. On the basis of the XRD analysis of resulting material, it was found that the aluminum ions have built into the crystallographic network of zinc oxide. Additionally, a formation of a spinel phase (ZnAl_2_O_4_) was found in the reaction product with an aluminum ion content of 15%.

SEM analysis showed that pure ZnO obtained in the microwave reactor formed hexagonal structures. Additionally, it formed “star-shaped” agglomerates. In samples with a 5% content of aluminum ions, the disruption of a hexagonal structure and the elongation of agglomerates occurred, while for the samples with an aluminum content of 15%, spherical agglomerates and the lack of a hexagonal structure were observed.

The synthesized zinc oxide nanopowders doped with aluminum ions were also investigated regarding their luminescence properties. All samples were excited with a 300 nm wavelength. In the range of 340 nm to 820 nm, none of the tested samples exhibited photoluminescence maxima. The quantum yield (QY) for synthesized samples was as follow (in respect to aluminum content): aluminum ion content (0–4.7%): QY = 1%, (1%): QY = 5.1%, (3%): QY = 6%, (5%): QY = 8.1%, (15%): QY = 9.2%. Based on the literature review, the luminescence performance of the obtained samples was found to be similar to values reported in scientific publications. The differences in QY values registered for samples in this study were related to the amount of doped aluminum ions [55,56].

X-ray diffraction (XRD) analysis showed that the obtained product was pure zinc oxide with aluminum ions embedded in the crystallographic network. This was evidenced by the change in lattice parameters. The presence of aluminum ions in the crystalline lattice was further justified by the density measurement. As the content of aluminum ions increased, the density of the nanopowder decreased. It was observed that for nanopowders obtained by the hydrothermal synthesis the decrease of the density values was rather mild. This was also registered in the case of changes of other lattice parameters, which increased mildly along with an increase of ion content. This was due to the fact that the solubility limit at high temperatures was lower than in hydrothermal synthesis. The aluminum ions probably ceased to integrate into the crystallographic network and were deposited on the surface of the powders. 

The use of a microwave reactor had shortened the reaction time and resulted in high purity of the synthesized particles. 

## Figures and Tables

**Table 1:** Analytical results for zinc oxide powders doped with Al(III) ions in 15 atm.

	Sample	Time [min]	Density [g/cm^3^]	BET [m^2^/g]	Average Grain size [nm]	Phase Composition
1	ZnO + 0.2%	5	5.33 ± 0.03	11 ± 1	93.67 ± 2.89	ZnO
	ZnO + 0.2%	10	5.41 ± 0.02	12 ± 1		ZnO
	ZnO + 0.2%	15	5.25 ± 0.02	12 ± 1		ZnO
2	ZnO + 0.5%	5	5.32 ± 0.03	27 ± 1	52.33 ± 6.89	ZnO
	ZnO + 0.5%	10	5.25 ± 0.02	19 ± 2		ZnO
	ZnO + 0.5%	15	5.34 ± 0.03	18 ± 2		ZnO
3	ZnO + 1%	5	5.30 ± 0.03	27 ± 2	42.33 ± 1.11	ZnO
	ZnO + 1%	10	5.24 ± 0.02	25 ± 2		ZnO
	ZnO + 1%	15	5.34 ± 0.03	25 ± 2		ZnO
4	ZnO + 3%	5	5.32 ± 0.03	36 ± 2	30.00 ± 0.67	ZnO
	ZnO + 3%	10	5.30 ± 0.03	37 ± 2		ZnO
	ZnO + 3%	15	5.34 ± 0.03	39 ± 2		ZnO
5	ZnO + 5%	5	4.57 ± 0.04	16 ± 2	53.33 ± 17.11	ZnO
	ZnO + 5%	10	5.16 ± 0.04	36 ± 3		ZnO + ZnAl_2_O_4_
	ZnO + 5%	15	5.20 ± 0.04	23 ± 3		ZnO + ZnAl_2_O_4_
6	ZnO + 15%	5	5.04 ± 0.06	61 ± 2	24.67 ± 7.56	ZnO + ZnAl_2_O_4_
	ZnO + 15%	10	5.03 ± 0.06	61 ± 3		ZnO + ZnAl_2_O_4_
	ZnO + 15%	15	5.14 ± 0.05	32 ± 2		ZnO + ZnAl_2_O_4_

**Table 2:** Value of lattice parameters depending on the content of Al(III) ions.

	Time (min)	Aluminum Ions Content (%_mol_)	Lattice Parameter a (Å)	Lattice Parameter c (Å)
0	5 10 15	0	3.251 ± 0.001 3.252 ± 0.002 3.251 ± 0.002	5.210 ± 0.001 5.211 ± 0.002 5.210 ± 0.002
1	5 10 15	0.2	3.252 ± 0.002 3.252 ± 0.002 3.251 ± 0.001	5.211 ± 0.002 5.210 ± 0.002 5.211 ± 0.001
2	5 10 15	0.5	3.255 ± 0.001 3.256 ± 0.002 3.256 ± 0.002	5.209 ± 0.001 5.208 ± 0.002 5.209 ± 0.002
3	5 10 15	1	3.253 ± 0.002 3.255 ± 0.002 3.257 ± 0.002	5.207 ± 0.002 5.206 ± 0.002 5.206 ± 0.002
4	5 10 15	3	3.250 ± 0.002 3.252 ± 0.002 3.257 ± 0.003	5.210 ± 0.002 5.211 ± 0.002 5.209 ± 0.002
5	5 10 15	5	3.251 ± 0.002 3.253 ± 0.001 3.251 ± 0.002	5.209 ± 0.002 5.209 ± 0.001 5.210 ± 0.002
6	5 10 15	15	3.250 ± 0.002 3.253 ± 0.002 3.251 ± 0.002	5.209 ± 0.002 5.209 ± 0.002 5.207 ± 0.002

## Data Availability

Not applicable.

## Supplementary Materials

The following supporting information can be downloaded at: https://www.mdpi.com/article/10.3390/ma15041403/s1 Figure S1. XRD patterns for selected samples obtained in the microwave reactor (10 min).
