# Supplementary material for: Luminescence Properties of Nano Zinc Oxide Doped with Al(III) Ions Obtained in Microwave-Assisted Hydrothermal Synthesis
Source: Materials (Basel). 2022 Feb 14;15(4):1403. doi: 10.3390/ma15041403 (PMC8877556; doi:10.3390/ma15041403)
Supplement: Supplementary file 1 [file materials-15-01403-s001.zip › materials-1576921-SI.pdf]

Supplementary Materials

Luminescence Properties of Nano Zinc Oxide Doped with Al(III) Ions Obtained in Microwave-Assisted Hydrothermal Synthesis

Tomasz Strachowski [1,*], Ewa Grzanka [2], Jan Mizeracki [2], Adrian Chlanda [1], Magdalena Baran [1], Marcin Małek [3], Klaudia Onyszko [3], Bartosz Januszewski [3] and Mirosław Przybysz [4]

[1] , Research Group of Graphene and Composites, Łukasiewicz Research Network–Institute of Microelectronics and Photonics IMiF; Al. Lotnikow 32/46, 02-668 Warsaw, Poland; adrian.chlanda@imif.lukasiewicz.gov.pl (A.C.); magdalena.baran@imif.lukasiewicz.gov.pl (M.B.)
[2] Institute of High Pressure Physics PAS "Unipress"; Sokolowska 29/37, 01-142 Warsaw, Poland; elesk@unipress.waw.pl (E.G.); janekm@unipress.waw.pl (J.M.)
[3] Faculty of Civil Engineering and Geodesy, Military University of Technology, ul. Gen. Sylwestra Kaliskiego 2, 00-908 Warsaw, Poland; marcin.malek@wat.edu.pl (M.M.); klaudia.onyszko@wat.edu.pl (K.O.); bartosz.januszewski@wat.edu.pl (B.J.);
[4] Institute of Robots Machine Design, Faculty of Mechanical Engineering, Military University of Technology, ul. Gen. Sylwestra Kaliskiego 2, 00-908 Warsaw, Poland; miroslaw.przybysz@wat.edu.pl
* Correspondence: tomasz.strachowski@imif.lukasiewicz.gov.pl

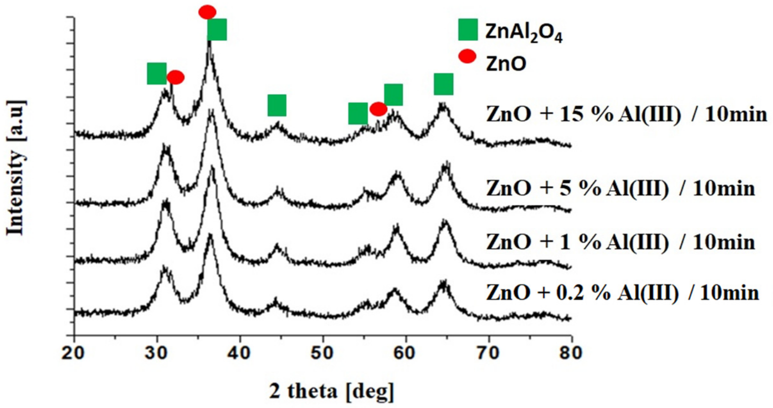

Figure S1. XRD paterns for selected samples obtained in microwave reactor (10 minutes).The plot shows the XRD peaks for selected ZnO samples doped with Al(III) ions obtained in a microwave reactor for 10 min. The appearance of spinel phase can be observed for samples with Al(III) ion content of 5 and 15 %. Below this value only ZnO phase was obtained.
